# Development and validation of blood-based diagnostic biomarkers for Myalgic Encephalomyelitis/Chronic Fatigue Syndrome (ME/CFS) using EpiSwitch^®^ 3-dimensional genomic regulatory immuno-genetic profiling

**DOI:** 10.1186/s12967-025-07203-w

**Published:** 2025-10-08

**Authors:** Ewan Hunter, Heba Alshaker, Oliver Bundock, Cicely Weston, Shekinah Bautista, Abel Gebregzabhar, Anya Virdi, Joseph Croxford, Ann Dring, Ryan Powell, Dominik Vugrinec, Caroline Kingdon, Carol Wilson, Sarah Dowrick, Jayne Green, Alexandre Akoulitchev, Dmitri Pchejetski

**Affiliations:** 1 Oxford BioDynamics Plc, Oxford, UK; 2 Norwich Medical School, University of East Anglia, Norwich, UK; 3 The London School of Hygiene & Tropical Medicine, London, UK; 4 Royal Cornwall Hospitals NHS Trust, Truro, UK

**Keywords:** Myalgic encephalomyelitis, Chronic fatigue syndrome, Autonomic dysregulation, ME/CFS, Diagnosis, Chromosome conformations, Epigenetics, Blood-based biomarkers, 3D-genomic profiling, Blood test

## Abstract

Myalgic Encephalomyelitis/Chronic Fatigue Syndrome (ME/CFS) is a debilitating, multifactorial disorder characterised by profound fatigue, post-exertional malaise, cognitive impairments, and autonomic dysfunction. Despite its significant impact on quality of life, ME/CFS lacks definitive diagnostic biomarkers, complicating diagnosis and management. Recent evidence highlights potential blood tests for ME/CFS biomarkers in immunological, genetic, metabolic, and bioenergetic domains. Chromosome conformations (CCs) are potent epigenetic regulators of gene expression and cross-tissue exosome signalling. We have previously developed an epigenetic assay, EpiSwitch^®^, that employs an algorithm-based CCs analysis. Using EpiSwitch^®^ technology, we have shown the presence of disease-specific CCs in peripheral blood mononuclear cells (PBMCs) of patients with amyotrophic lateral sclerosis (ALS), rheumatoid arthritis (RA), prostate and colorectal cancers, diffuse Large B-cell lymphoma and severe COVID-19. In a recent paper, we have identified a profile of systemic chromosome conformations in cancer patients reflective of the predisposition to respond to immune checkpoint inhibitors, PD-1/PD-L1 antagonists, with 85% accuracy. In this Retrospective case/control study (EPI-ME, *Epigenetic Profiling Investigation in Myalgic Encephalomyelitis)*, we used whole blood samples retrospectively collected from *n* = 47 patients with severe ME/CFS and *n* = 61 age-matched healthy control patients to perform whole-genome 3D DNA screening for CCs correlating to ME/CFS diagnosis. We identified a 200-marker model for ME/CFS diagnosis (Episwitch^®^CFS test). First testing on the retrospective independent validation cohort demonstrated a strong systemic ME/CFS signal with a sensitivity of 92% and a specificity of 98%.Pathways analysis revealed several likely contributors to the pathology of ME/CFS, including interleukins, TNFα, neuroinflammatory pathways, toll-like receptor signalling and JAK/STAT. Comparison with pathways involved in the action of Rituximab and glatiramer acetate (Copaxone) (therapies with potential in ME/CFS treatment) identified IL2 as a shared pathway with clear patient clustering, indicating a possibility of a potential responder group for targeted treatment.

**Supplementary Information:**

The online version contains supplementary material available at 10.1186/s12967-025-07203-w.

## Introduction

Myalgic Encephalomyelitis/Chronic Fatigue Syndrome (ME/CFS) is a debilitating, multifactorial disorder characterised by profound fatigue, post-exertional malaise, cognitive impairments, and autonomic dysfunction. ME/CFS affects millions worldwide, presenting with heterogeneous symptoms and unclear aetiology.

Despite its significant impact on quality of life, ME/CFS lacks definitive diagnostic biomarkers, complicating diagnosis and management. The absence of specific diagnostic tests necessitates reliance on clinical criteria, which can often lead to misdiagnosis or delayed diagnosis.

Recent research has focused on identifying molecular markers and developing blood-based diagnostic tests to improve clinical outcomes. There is accumulating evidence on molecular biomarkers for ME/CFS, highlighting advancements in immunological, genetic, metabolic, and bioenergetic domains (reviewed in [1]). Despite these advances, researchers have not yet established objective diagnostic tools or elucidated pathophysiological mechanisms.

The first hallmark of ME/CFS resulting from these studies is Immune dysregulation. Studies have reported altered cytokine profiles, including elevated pro-inflammatory cytokines such as interleukin-1β (IL-1β), IL-6, IL-10, tumour necrosis factor-alpha (TNF-α), and interferon gamma-induced protein 10 (IP-10) [1]. Additionally, increased expression of IL-8 and TNF-α suggests activation of the NF-κB signalling pathway, contributing to chronic inflammation [2]. Natural killer (NK) cell dysfunction, characterised by reduced cytotoxicity, has been observed in ME/CFS patients [3]. Autoantibodies targeting neurotransmitter receptors, including β2-adrenergic and muscarinic receptors, have also been identified, implicating autoimmunity in disease pathogenesis [4]. This was confirmed in a recent study where immune phenotyping of cerebrospinal fluid of ME/CFS patients revealed distinct immunotypes [5]. These findings overlap with post-COVID patients who showed upregulation of JAK/STAT signalling and a prolonged immune response [6].

The second hallmark of ME/CFS is transcriptional or epigenetic dysregulation in genes involved in immune response, energy metabolism, and neurotransmission. Notably, upregulation of purinergic receptors (P2 × 4, P2 × 5), transient receptor potential vanilloid 1 (TRPV1), and acid-sensing ion channel 3 (ASIC3) has been associated with post-exertional malaise [7]. DNA methylation changes have been implicated in ME/CFS. De Vega et al. identified over 12,000 differentially methylated sites in ME/CFS patients compared to healthy controls, mostly involving immune and metabolic pathways [8]. MicroRNAs (miRNAs), small non-coding RNAs regulating gene expression, have emerged as potential biomarkers. Altered expression of miR-21, miR-34a, miR-92a, miR-126, and miR-200c has been reported in ME/CFS patients [9]. Arcos-Burgos et al. showed that Neurodevelopment Genes Encoding Olduvai Domains Link ME/CFS to Neuropsychiatric Disorders [10].

The third hallmark of ME/CFS is reported disruptions in energy metabolism. Naviaux et al. reported abnormalities in metabolites related to the tricarboxylic acid (TCA) cycle, amino acid metabolism, and lipid metabolism, indicating a hypometabolic state [11]. Raman spectroscopy studies have demonstrated altered levels of aromatic amino acids (tryptophan, tyrosine, phenylalanine), glycerol, glycogen, and glucose in PBMCs of ME/CFS patients, reflecting impaired energy production and lipid metabolism [12]. Mitochondrial dysfunction has been implicated in ME/CFS pathophysiology. A nanoelectronics-based assay measuring cellular impedance in response to hyperosmotic stress revealed distinct patterns in ME/CFS patients, suggesting impaired cellular energy metabolism [13]. A recent large cohort study identified hundreds of metabolomic traits that differed significantly between cases and controls; however, single traits could not clearly distinguish cases from controls, and no combinatorial test was suggested [14].

Despite promising findings, several challenges hinder the clinical implementation of these biomarkers:

- **Heterogeneity**: ME/CFS exhibits diverse clinical presentations, complicating the identification of universal biomarkers.
- **Reproducibility**: Variability in study designs, sample sizes, and analytical methods affects the reproducibility of findings.
- **Validation**: Most studies require validation in larger, independent cohorts to confirm the diagnostic utility of proposed biomarkers.

Future research should focus on standardising methodologies, integrating multi-omics approaches, and conducting longitudinal studies to establish reliable, clinically applicable diagnostic tools.

While the reproducibility of findings between the included publications was limited, the majority of the studies validated the involvement of immune dysfunction in the pathology of ME/CFS and the use of PBMCs as a model to investigate the underlying pathology and the mechanism of illness.

Epigenetic changes are a powerful regulator of inflammatory and metabolic processes [15]. Chromosomal loops are the 3D chromatin structures that exist in all living cells and determine gene expression and genome network cross-regulation [16]. We have developed a novel epigenetic assay (EpiSwitch^®^ Explorer Assay), a bespoke design based on Agilent SurePrint 1 M array, that allows simultaneous screening for 10^6^ of 3D chromosomal conformations (CCs) in the circulating blood cells [17]. Using EpiSwitch^®^ technology, we have identified epigenetic signatures correlating to metabolic conditions and neuroinflammatory diseases such as amyotrophic lateral sclerosis (ALS) [18] and inflammatory conditions such as rheumatoid arthritis (RA) [19]. In two recent studies, we have identified chromosome conformations specific to the response to PD-1/PD-L1 immune therapy [20] and urothelial cancer.

Interestingly, although the 3D genomic regulatory architecture encompasses the whole genome, by mapping the top 3D genomic biomarkers to the closest genetic loci captured by their topological control (within 3Kb), it is possible to broaden the biological insights into genes, pathways and protein networks under the influence of 3D genomic regulation and associated cellular phenotype, contributing to the pathology of a disease and identify potential therapeutic strategies.

In a recent study, we have used EpiSwitch^®^ Explorer array platform and Machine learning algorithms to predict how individuals respond to COVID-19 infection. We have developed a blood-based prognostic test to predict the severity of infection, and 3D genomic markers identified potential treatments in biological pathways with direct relevance to immune system function, including T-cell signalling, macrophage-stimulating protein (MSP)-RON signalling, and calcium signalling [21]. EpiSwitch^®^-based commercial tests are now available to diagnose prostate cancer with 94% accuracy (PSE test) [22] and response to immune checkpoint inhibitors across 14 cancers with 85% accuracy (CiRT test) [20].

In this Retrospective case/control study (EPI-ME, Epigenetic Profiling Investigation in Myalgic Encephalomyelitis), we used blood samples from *n* = 47 patients with severe ME/CFS and *n* = 61 age-matched healthy controls. Whole Genome DNA screening for CCs correlating to ME/CFS diagnosis identified a 200-marker model for ME/CFS diagnosis (Episwitch^®^CFS test). Independent validation cohort testing demonstrated a remarkable sensitivity of 92% and specificity of 98% with overall diagnostic accuracy of 96% for ME/CFS diagnosis. Gene and pathway analysis revealed clear clustering in correlation to IL2, indicating a possibility of a potential responder group for targeted therapies.

## Materials and methods

### Patient characteristics

In this proof-of-concept retrospective case-control study, whole blood samples were obtained from the London School of Hygiene & Tropical Medicine Biobank for *n* = 47 patients with ME/CFS and *n* = 20 age-matched control patients. In addition, samples for *n* = 41 control patients were used from the OBD repository (Table 1). Of note, the imbalance of ME/CFS towards female patients reflects the proportional sex ratio in patients with severe ME/CFS.

Sample-size and power calculations using the automated sample size function with parameters (delta = 0.379, sigma = 0.3, power = 0.85), determined that 24 samples per group are sufficient to achieve 85% statistical power, which corresponds to detecting an effect size equivalent to a fold-change (FC) > 1.5 or an odds ratio (OR) > 1.2. The chosen validation sample (19 cases / 41 controls) was intended as a *moderate-sized independent cohort* to test generalisability and to obtain reasonably informative estimates of sensitivity and specificity, sufficient for an initial, proof-of-concept validation.

ME/CFS inclusion criteria: both biological sexes, age 20–80 years old, severe CFS – housebound.

ME/CFS exclusion criteria: any history of chronic illnesses, current or previous cancer, autoimmune conditions or any DNA-modifying or disease-modifying therapies or biological therapies. There was no available data regarding minor comorbidities and previous infection status, including glandular fever or COVID.

Controls Inclusion criteria: both biological sexes with a preferred equal distribution, and age 20–80 years old. Reasonable exercise tolerance, none of the four key CFS symptoms present or in the past. Preferably, an existing history of glandular fever or COVID.

Controls Exclusion criteria: any history of chronic illnesses, current or previous cancer, autoimmune conditions or any DNA-modifying or disease-modifying therapies or biological therapies.

The study was approved by the UK National Ethics Service Research Ethics Service (NRES), Research Ethics Committee (REC), and conducted in accordance with Good Clinical Practice guidelines and the Declaration of Helsinki. All participants provided written informed consent. All data were pseudo-anonymised. All procedures and protocols were performed in accordance with the relevant guidelines and regulations.

**Table 1:** Summary of clinical characteristics for patient cohorts used for biomarker discovery

Cohort	*N* (total)	Biobank	OBD	Male	Female	Age (mean)
**Control**	61	20	41	39	22	50
**ME/CFS**	47	47	0	8	39	45

### Preparation of 3D genomic templates

A 5 mL full blood sample was collected from ME/CFS patients and controls using BD Vacutainer^®^ plastic EDTA tubes. The tubes were frozen and stored at − 80 °C. Isolation of DNA from the whole cell lysate was performed as previously described [18], and DNA was fixed with formaldehyde. To identify interchromatin loops, fixed chromatin was digested into fragments with TaqI restriction enzyme, and the resulting DNA strands were joined, favouring cross-linked fragments. The cross-links were reversed, and PCR was performed using the primers designed using the algorithms of the EpiSwitch^®^ software (as described in detail in [18, 23–25]).

CC libraries were quantified using the Quant-iTTM Picogreen dsDNA Assay kit (Invitrogen) and normalised to 5 ng/µL. The EpiSwitch^®^ Explorer arrays were performed as published previously, with the modification of only one sample being hybridised to each array slide in the Cy3 channel. EpiSwitch^®^ Explorer arrays, based on the Agilent SureSelect array platform, allow for the highly reproducible, non-biased interrogation of ~ 1.1 million anchor sites for 3D genomic interactions (964,631 experimental probes and 2500 control probes).

### Custom microarray design

Custom microarrays were designed through the EpiSwitch^®^ software that uses a pattern recognition algorithm based on DNA sequence, which operates on Bayesian modelling and yields a probability score of whether a region is involved in long-range chromatin interactions. GRCh38 human genome assembly was annotated across ~ 1.2 million sites, and the potential to form long-range chromosome conformations [18, 19, 24–27]. The most probable interactions were identified and filtered on probabilistic score. Predicted interactions were limited to EpiSwitch^®^ sites larger than 10 kb and less than 300 kb apart. Repeat masking and sequence analysis were used to ensure unique marker sequences for each interaction. The EpiSwitch^®^ Explorer array (Agilent Technologies, Product Code X-HS-AC-02), containing 60-mer oligonucleotide probes, was designed to interrogate potential 3D genomic interactions. 964,631 experimental and 2,500 control probes were added to a 1 × 1 M CGH microarray slide design. The experimental probes were placed on the design in singlicate with the controls in groups of 250. The control probes consisted of six different EpiSwitch^®^ interactions generated during the extraction processes and used to monitor library quality. Four external inline control probe designs were added to detect non-human (*Arabidopsis thaliana*) spike-in DNA during the sample labelling protocol to provide a standard curve and control for labelling. The external spike DNA consists of 400 bp ssDNA fragments from genomic regions of *A. thaliana*. Array-based comparisons were performed as described previously, with the modification of only one sample being hybridised to each array slide in the Cy3 channel [18, 19, 24–27].

### Microarray statistical analysis

The cohorts of analysed samples were normalised by background correction and quantile normalisation, using the EpiSwitch^®^ R analytic package, which is built on the Limma Rank Product, tidyverse libraries. The datasets were combined into sample sets by processing batch. Data were corrected for batch effects using ComBat R script. The initial dataset was randomly partitioned into training and test sets using the createDataPartition function from the *caret* package in R. This partitioning was performed prior to any statistical analysis, ensuring that marker selection and model training were restricted to the training set only. The top 200 EpiSwitch^®^ CCS markers were identified exclusively within the training samples, and the predictive model was developed solely from these data. To minimise potential bias, the following safeguards were implemented. Randomisation: samples were randomised prior to analysis and balanced across batches to prevent systematic bias. Blinding: laboratory staff processing samples were blinded to clinical status. Batch correction: technical variation across processing runs was addressed using ComBat, applied after initial QC filtering. Independent validation: model performance was assessed not only on the internal test partition but also on independent external datasets, providing an unbiased measure of predictive accuracy. These steps were designed to reduce risk of data leakage and batch-related confounding, thereby ensuring that model performance reflects true biological signal rather than technical or procedural artifacts. Parametric (Limma R library, Linear Regression) and non-parametric (EpiSwitch^®^RankProd R library) statistical methods were performed to identify 3D genomic changes that demonstrated a difference in abundance between classes. The resulting data from both procedures were further filtered based on p-value and abundance scores (AS). Only 3D genomic markers with p-value < = 0.01 and AS (-1.2< ; >1.2) were selected. Both filtered lists from Limma and RankProd analysis were compared and the intersection of the two lists was selected for further processing.

### Machine learning and modelling

All analysis for this study was performed using libraries which are developed for the R Statistical Language (R version 4.2.0). Feature engineering of the EpiSwitch^®^Markers was performed using Recursive Feature Elimination (RFE) utilising Xgbtree, The XGBoost algorithm model70 was used for final test optimisation [28]. The best-performing XGBoost model was obtained with 50 boosting rounds (nrounds = 50) and shallow trees (max_depth = 3), using a moderate learning rate (eta = 0.6). Regularisation was implemented through a minimum loss reduction requirement (gamma = 0.5) and a low threshold for child node partitioning (min_child_weight = 0.5). To further limit overfitting, we incorporated partial feature sampling (colsample_bytree = 0.5) and row subsampling (subsample = 0.5). The grid search algorithm was used to optimise the hyperparameters and learning rate in each iteration. For drawing inferences, we used SHapley Additive exPlanations (SHAP) values that are computed by a game theoretical approach, which quantifies the contribution of each feature within a model to the final prediction of an observation.

### Genomic mapping

The 3D genomic markers from the statistically filtered list with the greatest and lowest abundance scores were selected for genome mapping. Mapping was carried out using Bedtools closest function for the 3 closest protein coding loci – upstream, downstream and within the long-range chromosome interaction (Gencode v33). All markers were visualized using the *EpiSwitch* Analytical Portal.

### Biological network/pathway analysis

Network analysis for functional/biological relevance of the 3D genomic markers was performed using the Hallmark Gene Sets and BioCarta and Reactome Canonical Pathway gene sets from the Molecular Signatures Database (MSigDB) [29]. Protein interaction networks were generated using the Search Tool for the Retrieval of Interacting Proteins (STRING) database [30].

## Results

### Identifying epigenetic biomarkers

We have performed whole-genome screening for potential ME/CFS biomarkers, comparing full blood samples from patients with severe ME/CFS (housebound) and healthy controls.

In contrast to prior studies in conditions such as prostate cancer, colorectal cancer, melanoma, ALS, and rheumatoid arthritis [18, 19, 22, 23, 25, 31, 32], which employed targeted or PCR-based approaches, we have based our ME/CFS model exclusively on the whole-genome array format. Rather than reducing the findings of a whole nucleome to 5–6 final markers transferred into a PCR readout, we looked for the established multi-marker footprints of ME/CFS on each tested individual. DNA microarray for 1 M CC markers was performed for each sample as described in Materials and Methods. This comprehensive approach allowed us to preserve the complexity of epigenetic signatures associated with ME/CFS, avoiding data overfitting at the development stage and enabling the detection of consistent patterns across patients.

**Fig. 1:**
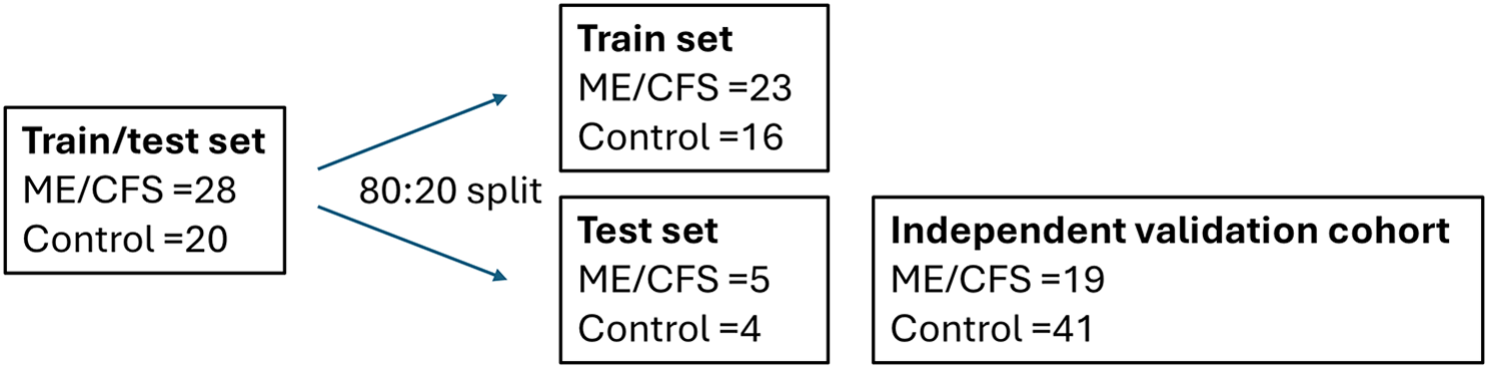
Train/test prediction model for identifying CCs corresponding to ME/CFS diagnosis

To ensure balanced sex representation, control and ME/CFS samples were rebalanced accordingly and subsequently divided into training and test sets using an 80:20 split (randomised with set.seed1999) (Fig. 1). The training set (*n* = 39) was used to identify the top 200 differentially expressed markers using both parametric and non-parametric approaches: linear modelling via limma and Rank Product analysis. Markers common to both methods were selected, and their statistical significance was ranked using the limma t-statistic. The resulting top 200 overlapping features were used to train an XGBoost classification model [28], employing the same training dataset.

**Fig. 2:**
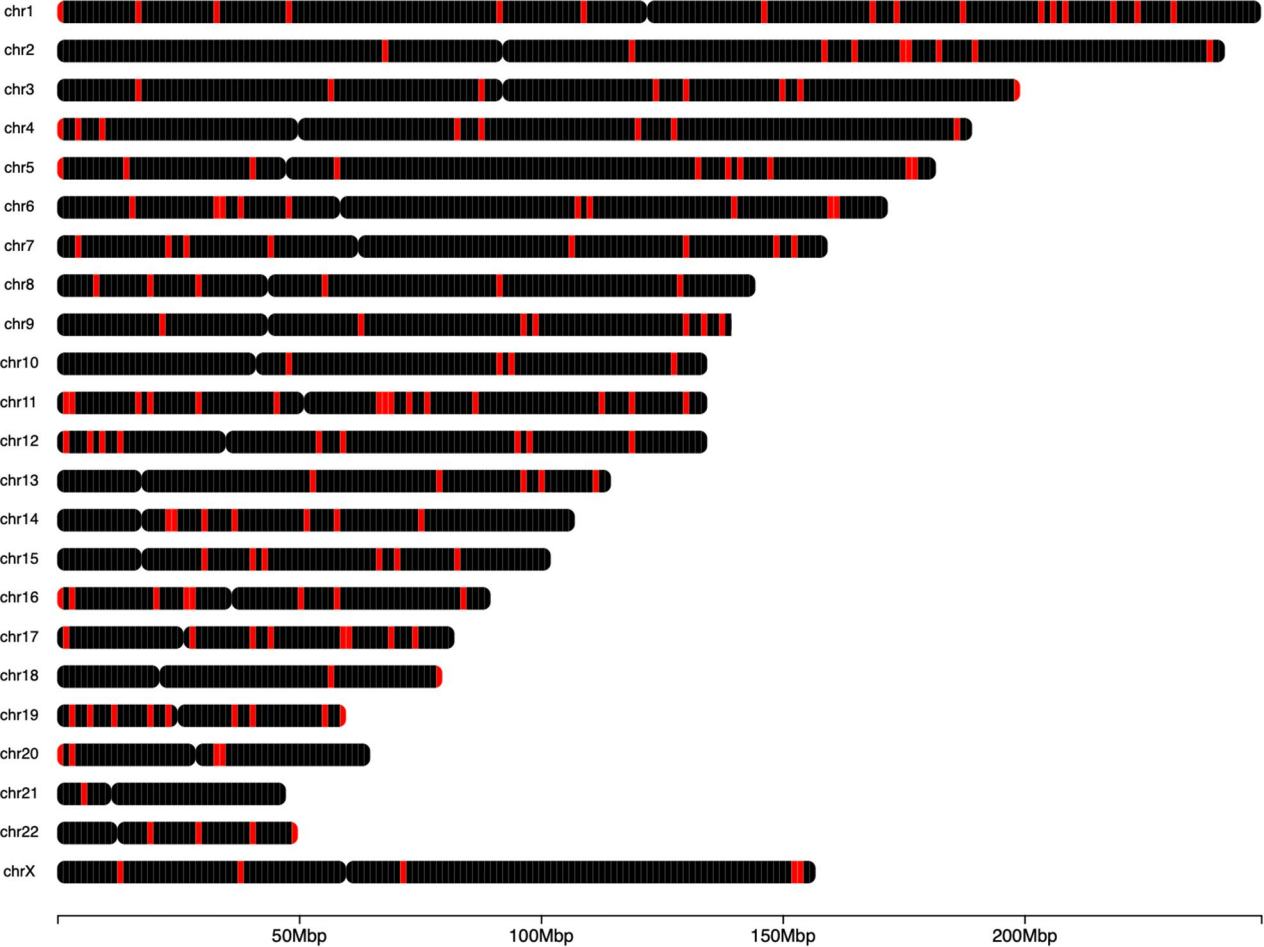
Whole genome mapping of top 200 CC markers. Red bars indicate mapped probes. Individual human chromosomes are shown on the y-axis (chr1-chr22 and the X chromosome). The heatmap shows the number of markers within a 0.3 Mb genomic window, with black representing a low density of markers and red indicating a high density of markers

Figure 2 demonstrates their mapping of the selected 200 markers to the human Genome.

The genetic locality of the top 10 markers/probes that mostly impacted on the model of the 200-marker set, is listed in Table S1.

These top-performing markers exhibited strong discriminatory power between ME/CFS patients and controls, suggesting their potential utility as diagnostic indicators. The probes were distributed across various chromosomes, indicating that ME/CFS may involve a complex interplay of genomic regions rather than a single locus of dysregulation.

24 ME/CFS and 45 control samples were set aside for a final independent validation cohort. These samples were not used at any point during the training phase, allowing for an unbiased evaluation of model performance. Test performance evaluation and diagnostic accuracy analysis showed a remarkable sensitivity of 92% and specificity of 98% with overall accuracy of 96% (Table 2). This high diagnostic accuracy underscores the strength of the selected epigenetic markers and the utility of the whole-genome approach in distinguishing ME/CFS from healthy phenotypes.

**Table 2:** Test performance evaluation and diagnostic accuracy on an independent validation cohort of 24 ME/CFS and 45 control patients

**Test**	**Present**	***n***	**Absent**	***n***	**Total**
Yes	True positive	22	False positive	1	23
No	False negative	2	True negative	44	46
**Total**		**24**		**45**	
**Statistic**		**Value (%)**	**95% Cl**,** %**		
Sensitivity		91.67	73-98.97		
Specificity		97.78	88.23–99.94		
Positive Likelihood Ratio		41.25	5.92-287.58		
Negative Likelihood Ratio		0.09	0.02–0.32		
Disease prevalence		34.78	23.71–47.21		
Positive Predictive Value		95.65	75.94–99.35		
Negative Predictive value		95.65	85.36–98.81		
Accuracy		95.65	87.82–99.09		

### Identified signalling pathways and their significance

**Fig. 3:**
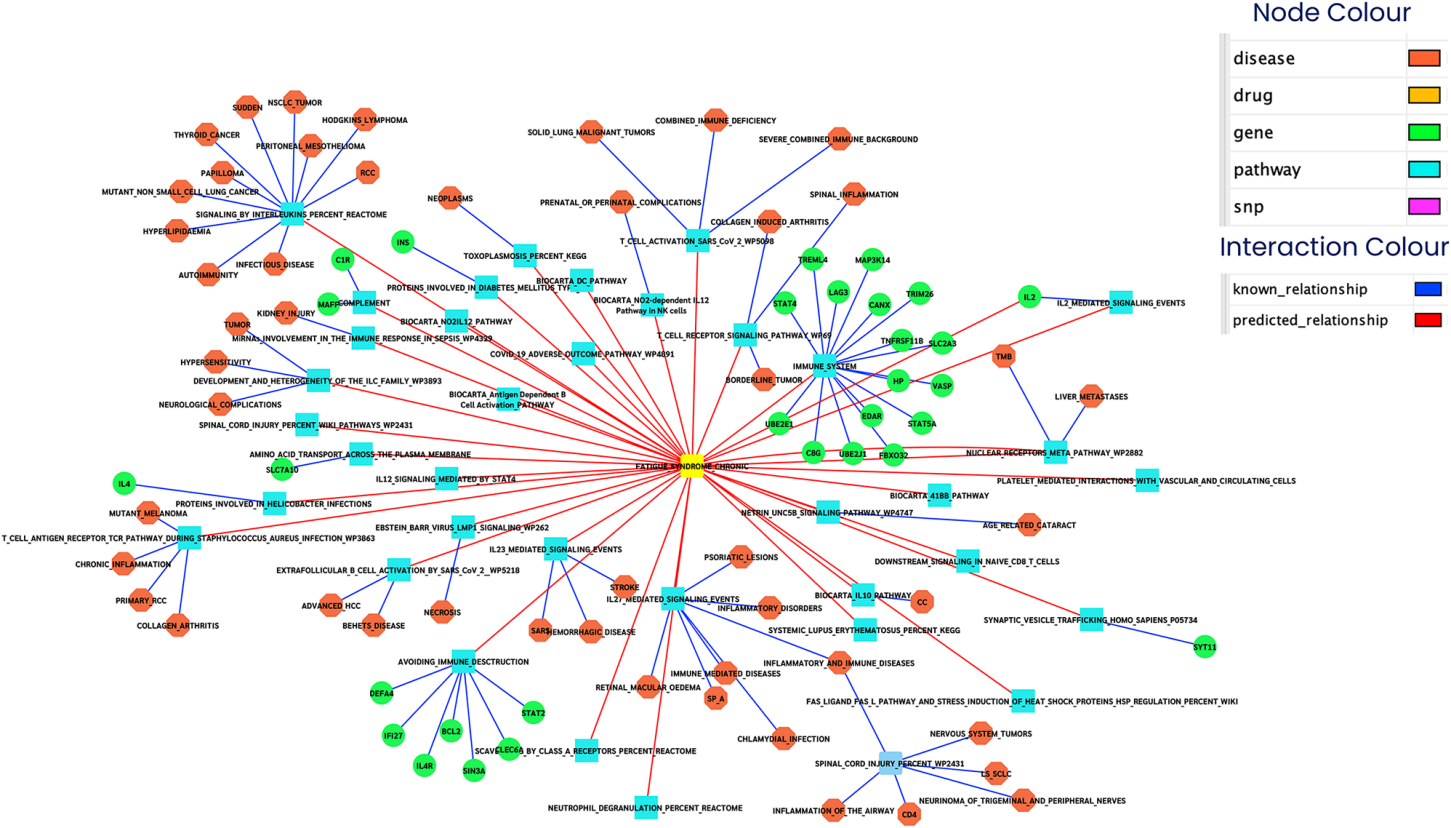
String map analysis of the protein–protein interaction network and signal transduction pathways related to the top 200 markers identified on the CC whole genome array in ME/CFS patients versus controls. Nodes represent proteins mapped from the nearest genes to each CCS; edges indicate high-confidence interactions (STRING confidence ≥ 0.8). The network demonstrates dense connectivity enriched for immune-regulatory and inflammatory pathways. Prominent hubs include IL-2, IL-10, TNFα, and TLR-related proteins, reflecting perturbations in cytokine signalling, innate immune sensing, and downstream JAK/STAT and NF-κB cascades

We mapped the top 200 predictive 3D-genomic markers from the EpiSwitch^®^ whole-genome screen to nearest genes and then performed protein–protein/network and pathway enrichment analysis (STRING, Reactome/MSigDB) [30]. The resulting network is densely connected and strongly enriched for immune and inflammatory signalling and cellular stress responses, consistent with an immune-dysregulation signal in ME/CFS (Fig. 3, Table S2).

**The key enriched pathways / network features include**:

Interleukin signalling (IL-2, IL-10 and related cascades). IL-2 emerged as a central hub in the STRING subnetwork: multiple top nodes connect to IL-2 signalling and T-cell regulation modules. IL-10 and other interleukins also feature prominently, suggesting dysregulated cytokine networks and altered T-cell/ regulatory-T cell balance. This converges with prior cytokine studies reporting altered circulating interleukins in ME/CFS [33].

TNF / NF-κB axis and innate inflammation. Several top markers map to genes participating in TNF signalling and NF-κB mediated innate responses, consistent with reports of elevated TNF and related inflammatory proteins in some ME/CFS cohorts. These pathways provide a plausible mechanistic link to systemic symptoms [34].

Toll-like receptor (TLR) signalling and innate immune sensors. TLR and MyD88-dependent modules are enriched, aligning with therapeutic and mechanistic interest in TLR3 (rintatolimod) and innate immune triggers in CFS. This supports a model in which altered innate sensing contributes to persistent immune activation [35, 36].

JAK/STAT signalling and cytokine-driven transcriptional programmes. JAK/STAT pathway genes are over-represented, consistent with the centrality of cytokine receptor signalling (IL-2, IL-6 families) in the network. JAK/STAT dysregulation is a shared theme across autoimmune/inflammatory diseases and provides a mechanistic bridge between cytokine changes and downstream transcriptional responses [37]. Overlap with autoimmune and neuroinflammatory diseases (MS, RA, SLE, etc.).

The ME/CFS network shows substantial overlap with pathways implicated in multiple sclerosis, rheumatoid arthritis and other chronic inflammatory disorders (shared nodes: IL-2, IL-10, CD4-T cell markers). This overlap is consistent with the concept of convergent immune pathways across neuroimmune and systemic inflammatory diseases, without implying identical aetiology [38, 39].

The resulting network revealed extensive connectivity between multiple cytokines (such as interleukins and TNF-α), inflammatory disorders (including rheumatoid arthritis, multiple sclerosis, and collagen-induced arthritis), and inflammatory pathways (including neuroinflammation, toll-like receptor signalling, and JAK/STAT). These results suggest a strong immunological component in ME/CFS pathology, with epigenetic alterations aligning closely with known pathways involved in chronic inflammation and immune dysregulation. There was also significant overlap with other diseases (such as Alzheimer’s, melanoma, non-Hodgkin’s lymphoma, ankylosing spondylitis, allergic disease, and psoriasis). This overlap highlights the possibility of shared epigenetic signatures or convergent pathways in chronic systemic and neuroinflammatory conditions.

It was previously shown that subsets of ME/CFS patients responded well to Rituximab [40]. Copaxone, a therapy for multiple sclerosis, was also suggested for ME/CFS treatment [41]. We have compared ME/CFS string networks to those of Rituximab and Copaxone (Figures S1, 2 and Tables S3, 4).

**Fig. 4:**
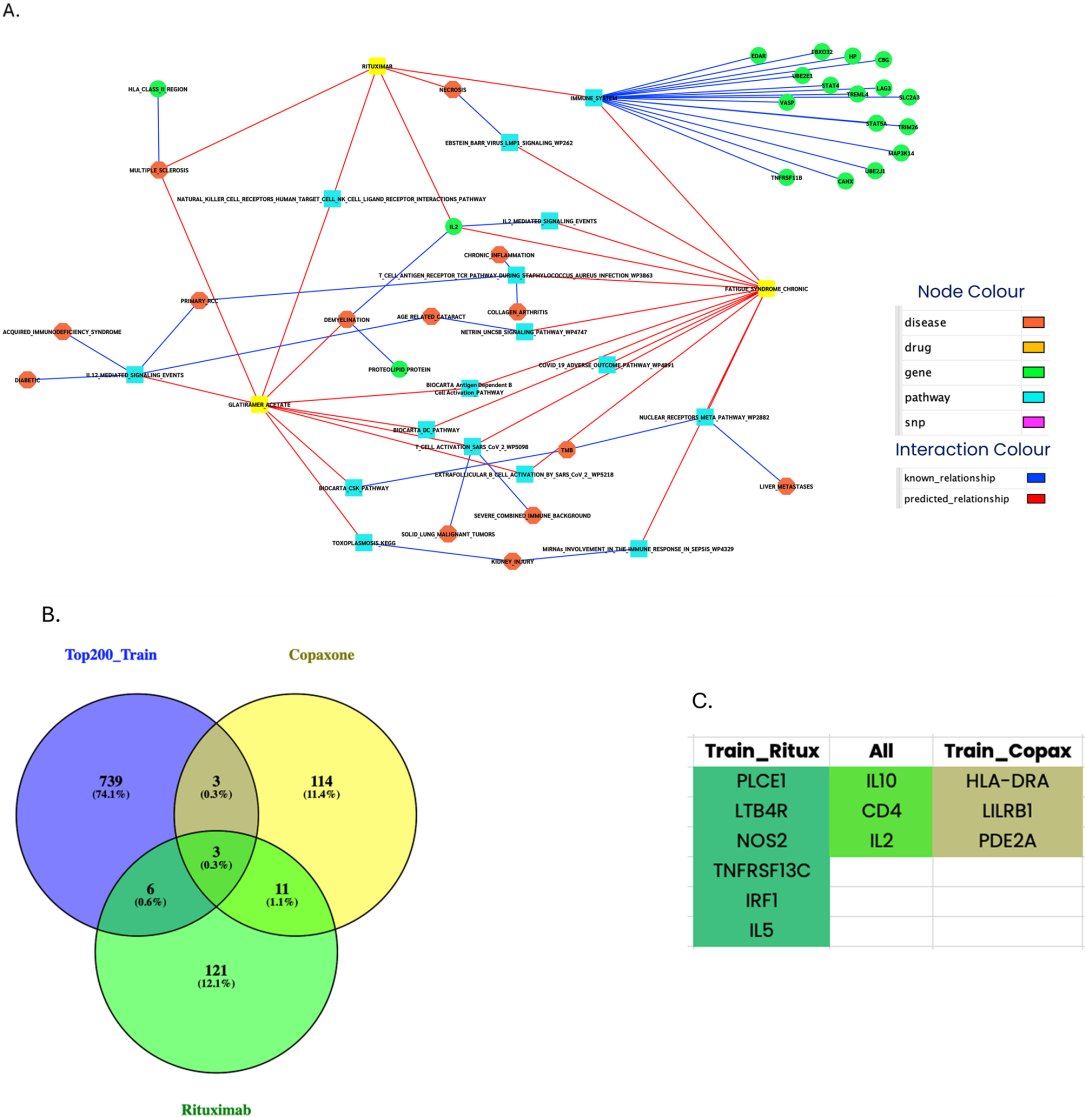
Pathway overlap between ME/CFS, Rituximab and Copaxone. (**A)** Genes identified using Episwitch^®^CFS screening and String pathway analysis. **(B)** The VENN diagram illustrates overlapping markers from the classifier EpiSwitch models, which are mapped to genes and then compared to the knowledge gene networks generated from Copaxone (Glatiramer Acetate) and Rituximab. **(C)** Names of shared genes between Rituximab, Copaxone and ME/CFS biomarker networks

The comparative analysis of the pathways showed significant overlap in IL10, IL2 and CD4 in all three networks, with more individual overlaps between ME/CFS and each therapy individually (Fig. 4).

This convergence supports the idea that epigenetic profiles in ME/CFS may inform therapeutic stratification and identify patients more likely to benefit from specific immunomodulatory treatments.

Of the three signalling molecules overlapping between ME/CFS, rituximab and Copaxone, we have chosen IL-2 as the major autocrine and paracrine T cell growth factor, which is, above all, responsible for the clonal expansion of antigen-specific T cells [42]. It also participates in the growth, differentiation, and activation of B cells, NK cells, and cytotoxic T cells and is involved in multiple proinflammatory conditions such as MS [43]. This central role of IL-2 in immune regulation and activation suggests its potential as both a biomarker and therapeutic target in ME/CFS.

**Fig. 5:**
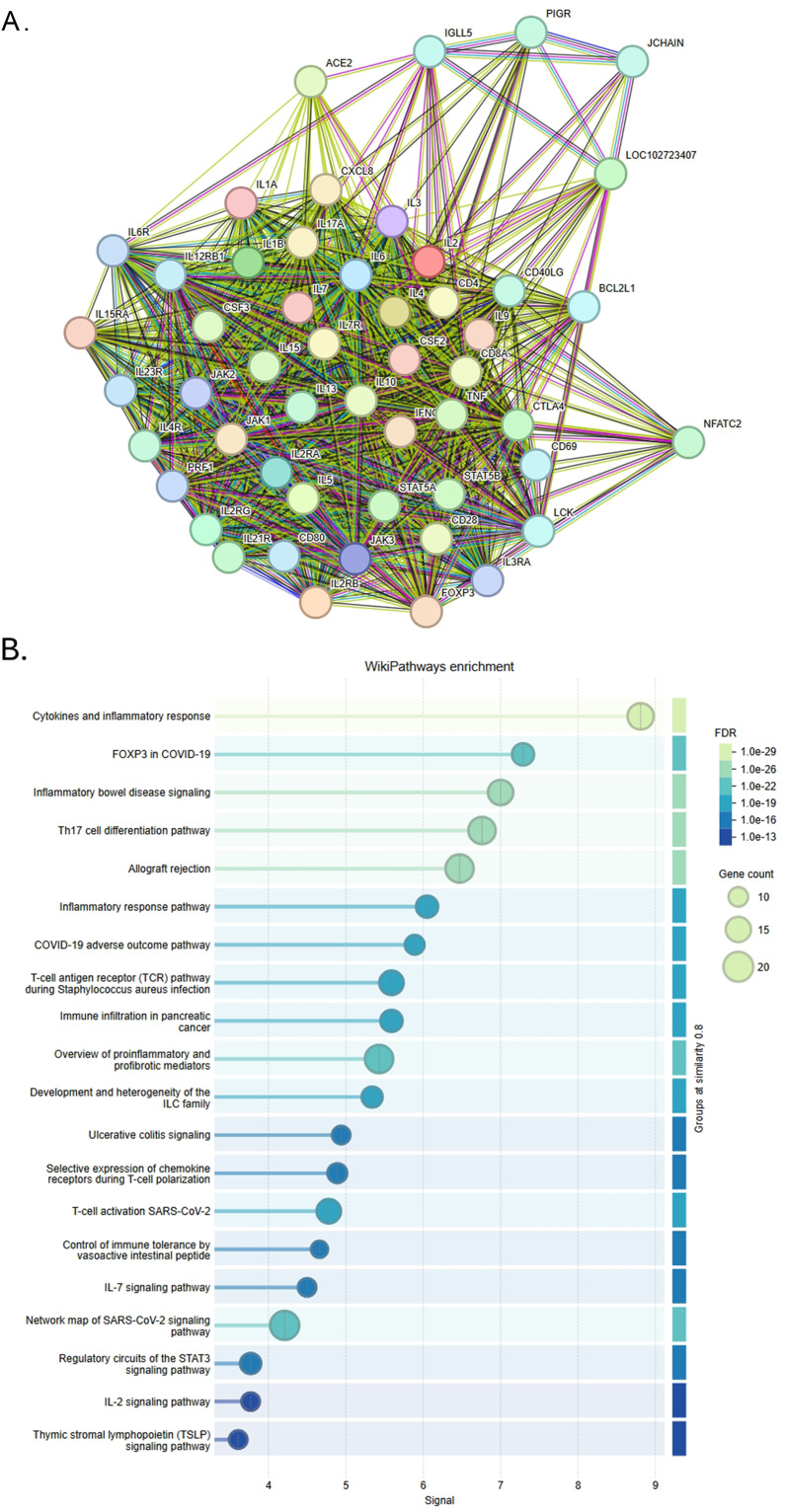
Top 50 STRING-connected Nodes in the IL-2-related pathways identified by String analysis (**A**) and Pathways Enrichment analysis (**B**). Pathway enrichment (Reactome and KEGG databases) of the top 50 CCS markers. Significantly over-represented pathways (FDR-adjusted *p* < 0.05), ranked by enrichment score. Key pathways include interleukin signalling (IL-2, IL-6, IL-10 families), T-cell activation and receptor pathways, inflammatory pathways, and FOXP3 in COVID-19 transcriptional responses

Figure 5 shows the top 50 STRING-connected Nodes in the IL-2-related pathways identified by String analysis (details of pathways in table S5). These nodes revealed tight clustering and robust interconnections, suggesting coordinated dysregulation of IL-2-mediated signalling cascades in ME/CFS patients.

### Biological interpretation & implications

#### Immune-centred signal

The pathway map points to coordinated disturbance of cytokine signalling, innate immune sensing and downstream JAK/STAT transcriptional programmes — a pattern compatible with (i) peripheral immune activation, (ii) altered T-cell regulation (IL-2/IL-10 axis), and (iii) neuroimmune cross-talk that can produce fatigue, cognitive symptoms and autonomic dysfunction described in ME/CFS [44].

#### Therapeutic stratification potential

Overlap with therapeutic networks (rituximab, glatiramer acetate/Copaxone) suggests a subset of patients whose epigenetic regulatory architecture reflects pathways targetable by B-cell depletion or T-cell-modulating agents — consistent with prior reports of heterogeneous rituximab responses in ME/CFS and the network overlap we observe [40].

#### Concordance with other omics

The pathway themes mirror findings from DNA-methylation and transcriptome studies in ME/CFS that implicate immune, metabolic and neuroendocrine genes, supporting the idea that 3D-genomic CCs capture a complementary regulatory layer linked to known disease biology. Methylation studies report immune-related DMPs and pathway enrichment [45].

**Fig. 6:**
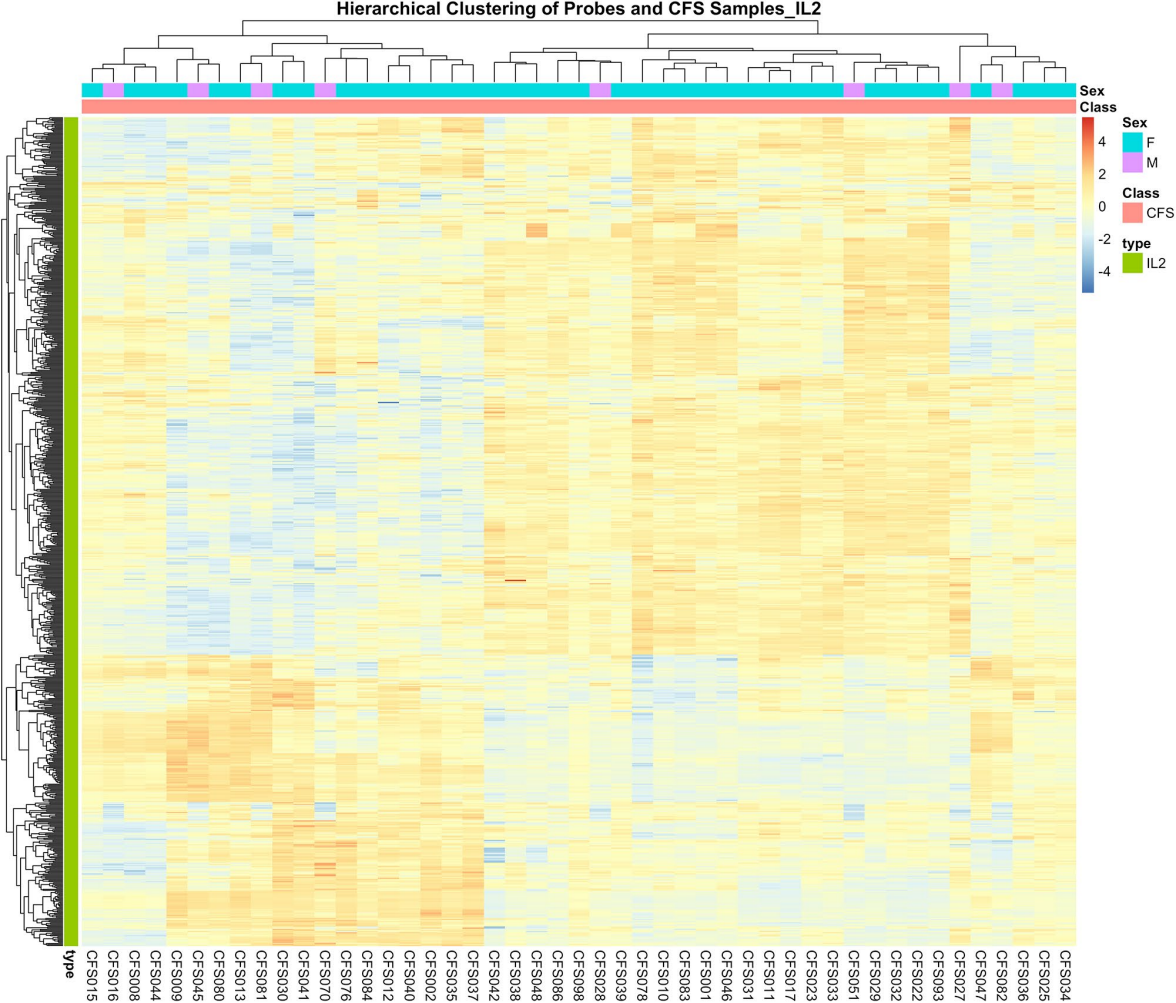
Hierarchical cluster of CFS patients and IL2-associated CCSs. Rows represent EpiSwitch markers associated with IL2, clustered according to similarity in marker profiles. Columns represent individual CFS patients, clustered based on their IL2 EpiSwitch signatures. Patient sex is indicated by the color-coded annotation bar above the heatmap, as shown in the legend

A differential response to Rituximab [40] In ME/CFS patients, highlighted the presence of cohorts of patients (~ 60% of the total number), who were more prone to respond. From the ME/CFS nucleome profile, we have selected the most significant CCs (765) that are associated with the 50 IL2 STRING genes/proteins. When analysed for these CCs, ME/CFS patients exhibited clear clustering with 18/29 division of the 47 patients analysed (Fig. 6).

This clear stratification indicates the potential for IL-2-related epigenetic markers to serve as a basis for patient subgrouping and personalized treatment approaches.

## Discussion

### Epigenetic and immunological changes in ME/CFS

Recent studies suggest that epigenetic dysregulation, particularly DNA methylation, may contribute to the complex pathophysiology of ME/CFS, influencing immune, metabolic, and neuroendocrine functions. Genome-wide methylation analyses using Illumina 450 K and EPIC arrays, as well as reduced representation bisulfite sequencing (RRBS), have identified thousands of differentially methylated positions (DMPs) in patients compared to healthy controls. Trivedi et al. identified over 17,000 DMPs across ~ 6,300 genes using the EPIC array, with altered promoter methylation in immune signalling genes [46]. Helliwell et al. utilised RRBS and found methylation differences in genes related to neuroimmune and metabolic regulation [45]. De Vega et al. examined glucocorticoid sensitivity and found over 12,000 DMPs, mostly hypermethylated in ME/CFS patients, particularly in metabolism-associated genes [8]. Importantly, this manuscript showed that methylation changes at immune and mitochondrial genes correlated with clinical symptoms and physical functioning scores (RAND-36), suggesting biological links to fatigue and post-exertional malaise. Dynamic methylation shifts during relapse and recovery phases in longitudinal ME/CFS cases implicated the contribution of stress and inflammatory response genes [47]. Gene-specific studies further reinforce these patterns. Hypomethylation at the *NR3C1* promoter (glucocorticoid receptor) has been repeatedly observed, implying HPA-axis dysregulation [48]. Other genes, such as *PRF1* (perforin) and *BDNF* (brain-derived neurotrophic factor), show altered methylation with potential relevance to immune function and neuroplasticity [49]. However, causality remains unresolved, and other non-DNA methylation markers (e.g., CCs, histone modifications, miRNAs) have not been well studied.

In this study, we have conducted a whole-genome CC screen to identify potential biomarkers for ME/CFS using whole blood samples from severely affected patients and matched healthy controls. Unlike our prior studies in other conditions that focused on a limited number of markers or employed PCR-based methods [18, 19, 22, 23, 25, 31, 32], this study utilised a comprehensive DNA microarray platform encompassing 1 million conformational capture (CC) markers, preserving the complexity of the epigenetic landscape. A robust five-step train/test predictive model employing XGBoost was applied (Fig. 1).

The top 200 probes identified through feature importance analysis were found to be distributed across multiple chromosomal regions, suggesting a polygenic signature of ME/CFS (Fig. 2).

Pathway enrichment using STRING analysis revealed significant involvement of immune and inflammatory signalling pathways, including interleukin cascades, TNF, Toll-like receptors, and JAK/STAT signalling. These pathways demonstrated substantial overlap with those implicated in other inflammatory and neuroimmune disorders such as multiple sclerosis, rheumatoid arthritis, and Alzheimer’s disease (Fig. 3).

Comparative network analysis revealed that the ME/CFS epigenetic signature shares key molecular nodes (e.g., IL-2, IL-10, CD4) with immunomodulatory therapies such as Rituximab and Copaxone, which have shown promise in ME/CFS treatment [41, 50].

IL-2 is a pro-inflammatory cytokine produced primarily by activated CD4 + T lymphocytes and plays a pivotal role in T-cell proliferation, survival, and differentiation [51]. It is crucial for regulatory T cell (Treg) maintenance and peripheral immune tolerance [52]. The expression of IL-2 by Th17 cells, lymphocytes with a key role in the pathogenesis of MS [53], was reported to be increased in the serum of patients with MS compared to healthy controls [54]. Higher IL-2 concentrations in CSF compared to controls were also observed [55], and found to be higher during a relapse of MS [56].

While the aetiology of ME/CFS remains elusive, multiple studies suggest that it is a neuroinflammatory condition (reviewed in [57]). Growing evidence implicates immune dysregulation, particularly involving cytokines such as Interleukin-2 (IL-2), in the pathogenesis of ME/CFS [58]. IL-2 may contribute to the neuroinflammation observed in ME/CFS. It can cross the blood-brain barrier and influence microglial activation, leading to central sensitisation and cognitive symptoms [59]. Animal studies support that peripheral IL-2 administration can induce fatigue-like behaviour and cognitive deficits, possibly via cytokine-induced sickness behaviour mechanisms [60].

### Therapeutic implications

Given IL-2’s dual pro-inflammatory and regulatory roles, modulating its signalling presents a therapeutic opportunity. Treatments aimed at dampening excessive IL-2-driven T-cell activation, such as immunomodulators or cytokine blockers, could be beneficial in subgroups with elevated IL-2 activity. However, clinical trials targeting IL-2 in ME/CFS are lacking, emphasising the need for personalised, biomarker-driven therapeutic approaches [61].

Several potential therapeutic candidates could fill this role. Patients receiving rituximab (a monoclonal antibody binding CD20) exhibited a significant decline in IL-2 and IFN-γ levels in peripheral blood, a substantial decrease in T-cell activation markers and inflammatory cytokine production, most prominent after repeated rituximab courses [62]. In patients with rheumatoid arthritis, rituximab significantly reduced serum concentrations of CRP, RF, anti-CCP, IL-2, IL-6, IL-7, IL-10, and ESR [63]. Similarly to Rituximab, Copaxone significantly reduced the percentage of IL-2-producing CD4 + and CD8 + T cells in patients with MS [64]. Kantegwa et al. showed that in MS patients Copaxone reduced intracellular synthesis of IL-2 and TNF-alpha by naive, memory and effector CD4(+)T cells [65]. The same group showed a significant decrease in the frequency of CD4^+^ T cells primed for secretion of IL-2 and TNF-α in patients treated with Copaxone [66].

Literature analysis suggests 12 potential therapies that may simultaneously reduce IL-2 and CD4 + cells and increase IL-10 (Table 3), ranging from biologics like Rituximab to small molecules like Tofacitinib. Several of these have already been trialled in ME/CFS or closely related conditions, though safety, tolerability, and immune impact remain critical considerations. Rituximab, a B–cell–depleting therapy, was evaluated in two Norwegian randomised controlled trials. Early results showed symptom improvement in some patients [40]. A larger phase III trial, however, failed to meet primary endpoints, though it confirmed the drug’s safety profile in ME/CFS patients [50]. A recent publication describing a follow-up of ME/CFS patients from two clinical trials of rituximab or cyclophosphamide showed that after six years, 44.1% of the cyclophosphamide group scored an SF-36 PF of at least 70, and 17.6% of at least 90, suggesting that cyclophosphamide in a subgroup may modulate the disease course in a beneficial way [67]. However, authors cautioned that cyclophosphamide has toxicity concerns and suggest that, rather than clinical implementation, these data should encourage efforts to better understand the disease mechanisms and to search for targeted and less toxic immune modulatory treatments.

Copaxone (glatiramer acetate) has not been formally trialled in ME/CFS, but its role in promoting regulatory T cells and IL-10 suggests potential utility [68]. Other agents like Rapamycin have shown promise. A recent study of Rapamycin showed significant improvement in fatigue and post-exertional malaise in ME/CFS patients [69].

Methotrexate, Azathioprine, and Mycophenolate Mofetil are well-established in autoimmunity and offer predictable suppression of IL-2 and CD4 + T cells. While not yet formally studied in ME/CFS, they may be repurposed with careful dosing and monitoring due to the known risks of infection and cytopenia.

Tofacitinib, a JAK inhibitor, has shown efficacy in inflammatory diseases like rheumatoid arthritis and ulcerative colitis [70]. Its ability to reduce IL-2 signalling and promote IL-10 makes it theoretically attractive for ME/CFS, but risks like thrombosis and viral reactivation would warrant caution.

Other low-risk options, such as Vitamin D3 and Hydroxychloroquine, offer safer starting points for trials, especially in mild or early-stage disease. Finally, Corticosteroids are known to provide short-term relief but are unsuitable for long-term use due to their side effect profile.

In conclusion, although not all 12 therapies have been trialled in ME/CFS, many have mechanisms that align with observed immune dysfunction. Future studies should focus on low-dose, precision immunomodulation with a careful eye on immunovigilance, infection risk, and subgroup stratification.

**Table 3:** 12 potential therapies that May simultaneously reduce IL-2 and CD4 + cells and increase IL-10

Therapy	Mechanism Summary	Effect on IL-2	Effect on CD4 + T Cells	Effect on IL-10	Clinical use / Notes
Rituximab	Anti-CD20 monoclonal antibody depletes B cells	Indirectly ↓ IL-2 (less T cell help)	↓ CD4 + T cell activation via B cell depletion	↑ IL-10 via regulatory B cells	RA, SLE, Non-Hodgkin’s Lymphoma (NHL), Chronic Lymphocytic Leukemia (CLL)
Copaxone (Glatiramer)	Shifts immune profile toward Th2/regulatory phenotype	↓ IL-2 production	↓ Inflammatory CD4 + T cells	↑ IL-10 from Tregs	MS
Rapamycin (Sirolimus)	mTOR inhibitor, blocks IL-2-mediated T cell proliferation	↓ IL-2 signaling	↓ CD4 + T cell proliferation	↑ IL-10 via Treg expansion	Autoimmune diseases, transplantation
Tacrolimus (FK506)	Calcineurin inhibitor, prevents IL-2 gene transcription	Strong ↓ IL-2	↓ CD4 + T cell activation	↑ IL-10 indirectly	Used in transplant and autoimmune therapy
Mycophenolate Mofetil	Inhibits purine synthesis, suppresses T and B cell proliferation	↓ IL-2 production	↓ CD4 + proliferation	↑ IL-10 (context-dependent)	Autoimmune and transplant medicine
Azathioprine	Purine analog, cytotoxic to proliferating T cells	↓ IL-2 production	↓ CD4 + proliferation	↑ IL-10 (indirectly)	SLE, IBD, autoimmune hepatitis
Tofacitinib	JAK inhibitor; blocks IL-2, IL-6, IFN signaling	↓ IL-2 signaling	↓ CD4 + activation, reduces inflammation	↑ IL-10 in innate and adaptive cells	RA, psoriatic arthritis, ulcerative colitis
Methotrexate (low-dose)	Anti-folate; reduces T cell activation and proliferation	↓ IL-2 transcription	↓ CD4 + T cell numbers	↑ IL-10 from T cells and monocytes	RA, psoriasis
Corticosteroids	Glucocorticoid receptor agonists, broad immunosuppression	Strong ↓ IL-2	↓ CD4 + T cells via apoptosis	↑ IL-10 transcription	Autoimmune/inflammatory diseases
Dimethyl Fumarate	Activates Nrf2, suppresses proinflammatory T cell pathways	↓ IL-2 production	↓ CD4 + activation	↑ IL-10 via immune modulation	MS, psoriasis
Hydroxychloroquine	Interferes with antigen presentation and TLR signaling	↓ IL-2 from T cells	↓ CD4 + activation	↑ IL-10 in macrophages and DCs	SLE, RA, Sjögren’s syndrome
Vitamin D3 (Calcitriol)	Hormonal immune modulator promoting regulatory immune tone	↓ IL-2 synthesis	↓ Th1 CD4+, ↑ Tregs	↑ IL-10 via Tregs	

A focused analysis of IL-2-associated pathways highlighted its central role in T cell regulation and immune activation. Hierarchical clustering based on IL-2-linked CCs demonstrated distinct patient subgroups, indicating the potential for biomarker-driven stratification and personalised therapeutic approaches in ME/CFS (Figs. 5 and 6).

### Diagnostic value

Several diagnostic approaches and tests have been previously suggested for ME/CFS (summarised in Table 4).

**Table 4:** Comparison of previously published ME/CFS diagnostic blood tests

Paper Title	Year Published	Diagnostic Accuracy	Method	*Limitations*
Discriminative Validity of Metabolic and Workload Measurements for Identifying People With Chronic Fatigue Syndrome [71].	2013	95.1% accuracy	Exercise and Gas Exchange Data	Needs hospital adaptation
Bottom-up proteomics suggests an association between differential expression of mitochondrial proteins and chronic fatigue syndrome [72].	2016	sensitivity 85%, specificity 72%. AUC 0.793	Nano-liquid chromatography electrospray ionization mass spectrometry	Clinical test adaptation required
A nanoelectronics-blood-based diagnostic biomarker for myalgic encephalomyelitis/chronic fatigue syndrome (ME/CFS) [13].	2019	n/a	Blood cells impedance	Time, expense and expertise
Assessing cellular energy dysfunction in CFS/ME using a commercially available laboratory test [73].	2019	n/a	Mitochondrial energy score	Poor reliability reported
Identification of actin network proteins, talin-1 and filamin-A, in circulating extracellular vesicles as blood biomarkers for human myalgic encephalomyelitis/chronic fatigue syndrome [74].	2019	AUROC for circulating EVs was 0.802	Actin network proteins in circulating extracellular vesicles	Further validation needed
Profile of circulating microRNAs in myalgic encephalomyelitis and their relation to symptom severity, and disease pathophysiology [75].	2020	90% accuracy	Circulating microRNAs	Eleven microRNAs, clinical adaptation
Cell-Based Blood Biomarkers for Myalgic Encephalomyelitis/Chronic Fatigue Syndrome [76].	2020	89% sensitivity 77% specificity	mTORC1 activity and PBMC viability	Requires manual counting and microscopy
Plasma proteomic profiling suggests an association between antigen driven clonal B cell expansion and ME/CFS [77].	2020	AUCs of 0.774–0.838	plasma proteomic profiling via UPLC-MS/MS	Clinical test adaptation required
Multimodal MRI of myalgic encephalomyelitis/chronic fatigue syndrome: A cross-sectional neuroimaging study toward its neuropathophysiology and diagnosis [78].	2022	n/a	Multimodal MRI	Requires further development
Diagnosis of Myalgic Encephalomyelitis/Chronic Fatigue Syndrome With Partial Least Squares Discriminant Analysis: Relevance of Blood Extracellular Vesicles [79].	2022	AUC = 0.71	PBMC and Extracellular Vesicle miRNAs	Limited diagnostic power of individual or combined miRNA .
Proteomics and cytokine analyses distinguish myalgic encephalomyelitis/chronic fatigue syndrome cases from controls [80].	2023	79,1% accuracy	Proteomics multiplex assay	Less accuracy than other studies
Developing a Blood Cell-Based Diagnostic Test for Myalgic Encephalomyelitis/Chronic Fatigue Syndrome Using Peripheral Blood Mononuclear Cells [12].	2023	91% accuracy	Raman spectroscopy of PBMCs	Specific single-cell Raman platform
Deficient butyrate-producing capacity in the gut microbiome is associated with bacterial network disturbances and fatigue symptoms in ME/CFS [81].	2023	n/a	Microbiome	Requires further development
Discriminating Myalgic Encephalomyelitis/Chronic Fatigue Syndrome and comorbid conditions using metabolomics in UK Biobank [82].	2024	83% accuracy	Metabolomics using blood plasma NMR profiles	NMR translation to hospital setting

The field of blood biomarkers for ME/CFs was recently comprehensively reviewed by Clarke et al. [83]. It was noted by the authors that while ‘proposed findings hold promise as potential blood-based quantitative diagnostic biomarkers for ME/CFS, further research is required to determine their specificity to ME/CFS and adoptability for clinical use’. While some studies achieved 90% accuracy, the proposed methods required extensive time, expense and expertise that is beyond current clinical settings. Indeed, while some of the suggested methods achieved ~ 90% accuracy, they required complex laboratory workflows.

Using an independent validation cohort, the whole DNA approach achieved high classification performance with 92% sensitivity, 98% specificity, and 96% overall accuracy for the detection of ME/CFS (Table 2), Episwitch^®^CFS test. This high diagnostic accuracy underscores the strength of the selected epigenetic markers and the utility of the whole-genome approach in distinguishing ME/CFS from healthy phenotypes. Based on the observed effect sizes from our independent validation cohort (accuracy = 96%, sensitivity = 92%, specificity = 98%), the study achieves > 90% power to detect diagnostic performance above 85% at α = 0.05. We acknowledge that larger, multi-centre cohorts are needed to confirm generalisability.

In contrast to previously published tests, EpiSwitch^®^ has already been translated to clinical diagnostics (e.g., prostate cancer), making it more scalable for clinical adoption. The EpiSwitch^®^ regulatory genome architecture platform has already demonstrated successful translation into clinical diagnostics, distinguishing it from exploratory biomarker modalities [84]. The technology operates under ISO 13,485, ISO 9001, and ISO 15,189 standards in CLIA- and UKAS-accredited laboratories, ensuring assay reproducibility, stability, and scalability. Examples of translation include the **EpiSwitch PSE test for prostate cancer**, which achieved 94% accuracy and reduced unnecessary biopsies by 79% in prospective and real-world studies, and the **CiRT test for immunotherapy response**, which predicts PD-1/PD-L1 response with 85% accuracy, outperforming standard PD-L1 IHC [20, 22, 85]. Both tests are now commercially available in the US (CPT PLA codes 0433U and 0332U) and UK, with established reimbursement and widespread clinical uptake. On this foundation, the current ME/CFS biomarkers can follow a clear path to clinical translation, supported by further multi-center validation.

Our epigenetic chromosome conformation blood test demonstrated a high diagnostic sensitivity and specificity, surpassing the performance of previously published biomarker-based approaches. In comparison to previous studies, our study benefits from a larger cohort of patients with a homogenous level of fatigue. Importantly, our use of a whole-genome array allowed us to capture the broad, systems-level epigenetic dysregulation associated with ME/CFS, rather than focusing on individual pathways or isolated biomarkers. This comprehensive view reflects the underlying complexity and heterogeneity of disease-related gene regulation. Our findings suggest that chromatin architecture may be a more stable and disease-specific marker in ME/CFS, potentially offering a more reliable, reproducible, and clinically actionable diagnostic platform.

Although the EpiSwitch^®^ CFS test showed high accuracy in distinguishing ME/CFS from healthy controls, its performance against other chronic inflammatory diseases has not yet been tested. STRING network analysis indicated overlapping pathways with conditions such as MS and RA, suggesting possible shared epigenetic architecture. Future work with external disease cohorts will be essential to confirm disease specificity and refine biomarker panels capable of discriminating ME/CFS from other systemic inflammatory or autoimmune disorders. Given that our cohort was restricted to severe, housebound patients, the applicability of these biomarkers to moderate or mild ME/CFS remains to be investigated in future studies. Although we employed independent training and validation subsets, both were derived from overlapping biobank sources. Larger-scale, prospective validation in multi-centre cohorts will be essential to establish external validity. The observed epigenetic changes may be downstream of chronic immune activation; longitudinal and interventional studies (e.g., before/after immunomodulatory therapy) are needed to test causality.

## Conclusion

This study applied a genome-wide epigenetic screening approach using CC microarray profiling to identify ME/CFS biomarkers from blood samples of severely affected patients.

The top 200 predictive probes mapped broadly across the genome and revealed key epigenetic patterns associated with immune dysregulation. STRING analysis of these markers identified enriched pathways involving cytokines (e.g., IL-2, TNF), inflammatory signalling (e.g., TLR, JAK/STAT), and overlap with other chronic inflammatory diseases.

IL-2 emerged as a central node linking ME/CFS to therapeutic networks of Rituximab and Copaxone. Hierarchical clustering based on IL-2-associated markers showed distinct patient subgroups, supporting potential for precision diagnostics and targeted interventions. Targeted modulation of IL-2 pathways may offer novel therapeutic strategies for ME/CFS, especially in immunologically defined subtypes.

A five-step XGBoost-based machine learning model, validated across independent cohorts, achieved 92% sensitivity and 98% specificity in diagnosing ME/CFS (Episwitch^®^CFS test).

## Supplementary Information

Below is the link to the electronic supplementary material.

Supplementary Material 1: Figure S1. String map analysis of the signal transduction pathways related to the Copaxone Knowledge Network (Pathways & Disease).

Supplementary Material 2: Figure S2. String map analysis of the signal transduction pathways related to the Rituximab Knowledge Network (Pathways & Disease).

Supplementary Material 3: Table S1. Genetic locality of the top 10 markers/probes that most impacted the model of the 200-marker set.

Supplementary Material 4: Table S2. String analysis of signalling pathways related to the top 200 markers identified on the CC whole genome array.

Supplementary Material 5: Table S3. String analysis of signalling pathways overlapping between the top 200 markers identified on the CC whole genome array and Rituximab-related pathways.

Supplementary Material 6: Table S4. String analysis of signalling pathways overlapping between the top 200 markers identified on the CC whole genome array and Copaxone-related pathways.

Supplementary Material 7: Table S5. Top 50 STRING-connected Nodes in the IL-2-related pathways.

## Data Availability

Knowledge graphs were generated using the EpiSwitch 3D-Genomics large language model (LLM), which employs a unique embedding approach. This platform integrates advanced semantic parsing with over 1.5 billion experimentally-verified 3D genome interactions. Leveraging proprietary AI models trained on chromatin architecture, it enables mapping of regulatory circuitry, identification of causal mechanisms, and prediction of the effects of non-coding and structural variants within their native 3D genomic context. These tools are deployed on Google Cloud in collaboration with Google. Data is available on request https://www.oxfordbiodynamics.com/contact-us#contact-us-form-wrapper.
