# Supplementary figures and images for: Development and validation of blood-based diagnostic biomarkers for Myalgic Encephalomyelitis/Chronic Fatigue Syndrome (ME/CFS) using EpiSwitch® 3-dimensional genomic regulatory immuno-genetic profiling
Source: J Transl Med. 2025 Oct 8;23:1048. doi: 10.1186/s12967-025-07203-w (PMC12506310; doi:10.1186/s12967-025-07203-w)

## Slide 1
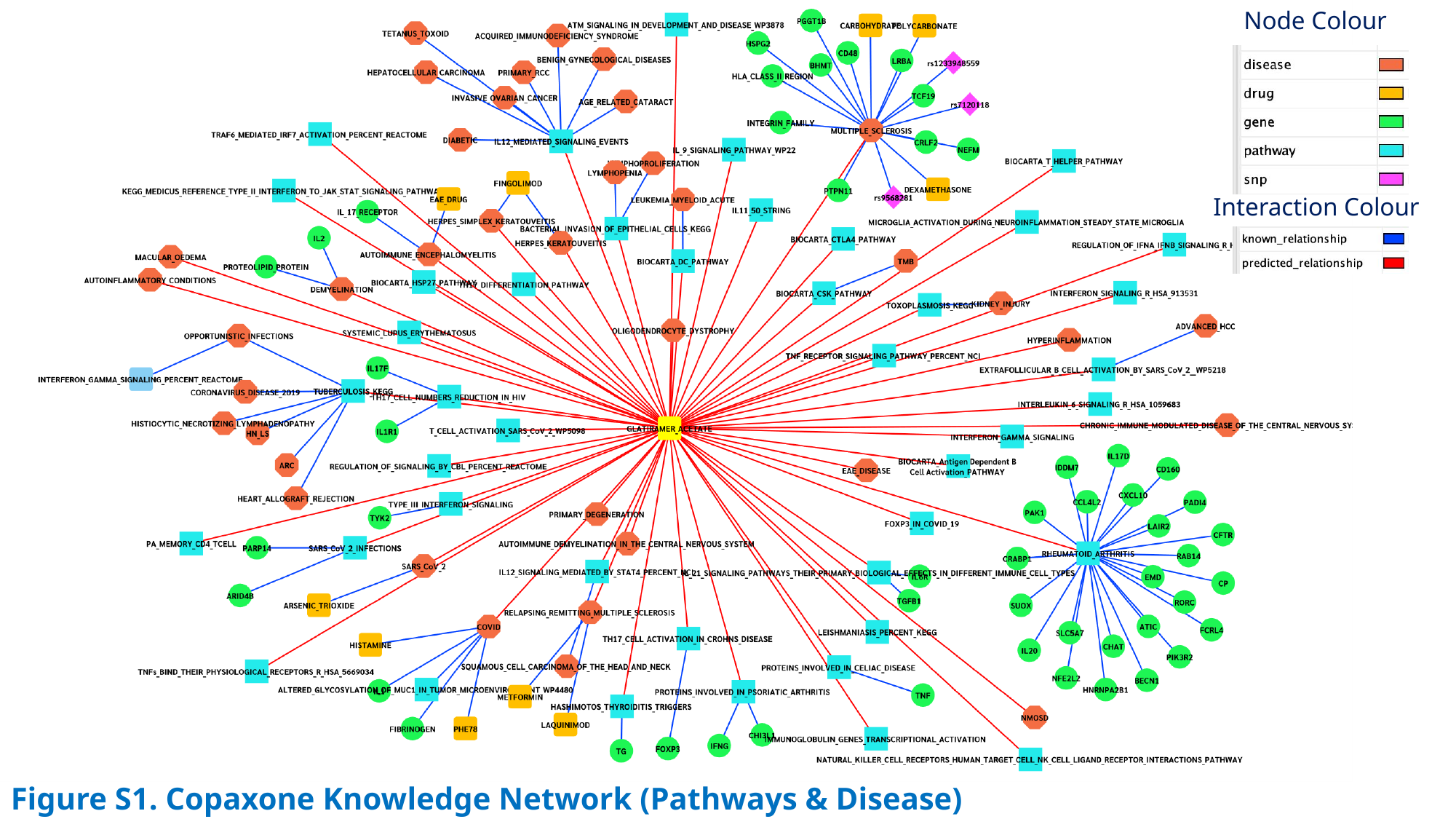

Node Colour
Interaction Colour
Figure S1. Copaxone Knowledge Network (Pathways & Disease)

Supplement: Supplementary file 1 — Supplementary Material 1: Figure S1. String map analysis of the signal transduction pathways related to the Copaxone Knowledge Network (Pathways & Disease). [file 12967_2025_7203_MOESM1_ESM.pptx]

## Slide 1
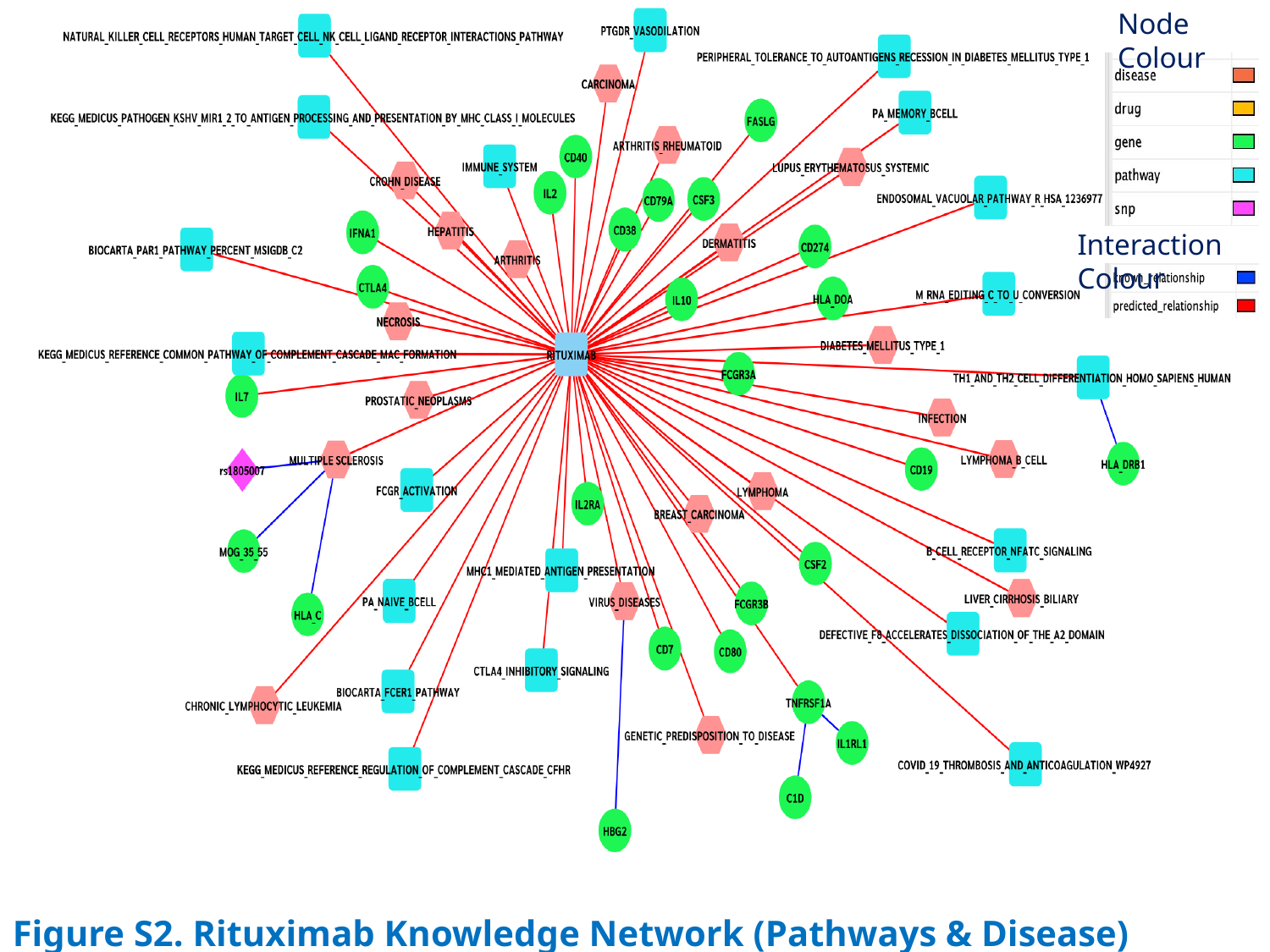

Node Colour
Interaction Colour
Figure S2. Rituximab Knowledge Network (Pathways & Disease)

Supplement: Supplementary file 2 — Supplementary Material 2: Figure S2. String map analysis of the signal transduction pathways related to the Rituximab Knowledge Network (Pathways & Disease). [file 12967_2025_7203_MOESM2_ESM.pptx]
